# Editorial: Fruits, vegetables and herbs: medicinal chemistry, metabolic and health effects

**DOI:** 10.3389/fnut.2023.1225577

**Published:** 2023-07-12

**Authors:** Branca M. Silva, Luís Rato, Cátia V. Vaz, Ana Vinha, Maria B. P. P. Oliveira

**Affiliations:** ^1^University of Beira Interior, Covilhã, Portugal; ^2^(CICS-UBI) Health Sciences Research Centre, University of Beira Interior, Covilhã, Portugal; ^3^Health School of the Polytechnic Institute of Guarda, Guarda, Portugal; ^4^Department of Chemical Sciences, Faculty of Pharmacy of University of Porto, REQUIMTE/LAQV, Porto, Portugal; ^5^FP-ENAS (UFP Energy, Environment and Health Research Unit), CEBIMED (Biomedical Research Centre), University Fernando Pessoa, Porto, Portugal

**Keywords:** fruits, vegetables, herbs, obesity, infertility, cancer, diabetes, cardiovascular diseases

Non-communicable diseases (NCD), including cardiovascular diseases, cancers, chronic respiratory diseases and diabetes, are recognized as a major global challenge in the United Nations 2030 Agenda for Sustainable Development. Unhealthy lifestyles, namely physical inactivity, smoking, alcohol abuse, and unbalanced eating habits, are contributing to the spread of these chronic diseases (1).

A plant-based diet (PBD) is any dietary pattern that emphasizes the consumption of foods derived from plants and excludes or limits the intake of most or all animal products (2). Traditional Mediterranean and Eastern diets are good examples of healthful and sustainable plant-based dietary patterns. In fact, accumulating data strongly indicate that the high consumption of fruits, vegetables, herbs (medicinal and aromatic plants), legumes, whole grains, nuts, and olive oil is associated with a lower risk of morbidity, disability, and mortality by NCD (1, 2).

Fruits, vegetables and herbs play an important role in the quality of PBD once they are characterized by a nutrient and phytochemical profile that is low in calories and high in fibers, vitamins, flavonoids, phenolic acids, glucosinolates, terpenes, sterols, and methylxanthines, with strong antioxidant, hypoglycemic, anti-inflammatory, anti-hyperlipidemic, anti-hypertensive, neuroprotective, and anticarcinogenic properties (2–6). The evidence suggests that the health benefits of consuming these plant-based foods are due to additive and synergistic interactions between their different phytocomponents.

Considering that the selection of a healthy, well-balanced and sustainable dietary pattern by the general population is closely linked to their literacy in terms of nutrition knowledge, the main goal of our Research Topic “*Fruits, Vegetables and Herbs: Medicinal Chemistry, Metabolic and Health Effects*” is to present an overview of the possible role of fruits, vegetables and herbs (and/or their phytochemicals) in the prevention and management of NCD.

Our Research Topic is composed by nine papers (five original researches, one mini-review and three systematic reviews) covering several research aspects and recent advances related to the different classes of nutrients/phytochemicals commonly found in fruits, vegetables and herbs, highlighting their chemical structures, occurrence, biological importance and mechanisms of action/interaction.

Globally, cardiovascular diseases are the leading cause of mortality, followed by cancers (1). Hypertension, hyperlipidemia, hyperglycaemia and oxidative stress are key metabolic changes that increase the cardiovascular risk. Herein, Feng et al. evaluated the effect of the oral administration of a hawthorn fruit hydroalcoholic extract in the prevention of the progression of hyperlipidemia in a high-fat diet rat model. In addition, Sookying et al. summarized and discussed the botanical aspects, phytochemical profile, antioxidant activity and toxicity of the leaves of tamarind, an African tropical food and medicinal plant.

Carotenoids are tetraterpene antioxidant pigments that are widely spread in colored fruits and vegetables, exhibiting yellow, orange, or red colors. Obesity is a triggering factor for several human diseases, namely for non-alcoholic fatty liver disease (NAFLD). In our Research Topic, Balbuena et al. evaluated and compared the effects of supplementation with orange carrots (carotenoid-rich) and white carrots (carotenoid-deficient) on NAFLD progression in a high-fat diet induced obese mice model.

Overweight/obesity is also an important risk factor for diabetes associated with insulin resistance. Considering that resveratrol is a polyphenol with antioxidant, hypoglycaemic, anti-inflammatory, and anti-hyperlipidemic properties, and the close relationship between insulin resistance and polycystic ovary syndrome (PCOS), Liang et al. reported the beneficial effect of resveratrol administration on ovarian insulin sensitivity in a PCOS rats model.

B-type procyanidins are polyphenolic compounds commonly found in Rosaceae family fruits, such as apples, pears and quinces (7). In our Research Topic, Osakabe et al. summarized the human intervention trials on the hormetic responses induced by this procyanidins type that may be responsible for the health effects of pome fruits intake. They also discussed the hypothesis of this hormetic pattern arises via neurotransmitter receptors expressed in the central nervous system.

Aging is the primary risk factor for most neurodegenerative diseases and Parkinson's disease (PD) is the second most common (after Alzheimer's disease) (3, 4). PD is characterized by the loss of dopaminergic neurons that causes involuntary/uncontrollable movements, rigidity, disequilibrium and lack of coordination. Li et al. reported the multiple mechanisms of action of Ping-wei-san plus herbal decoction (a traditional Chinese medicinal product composed by a mixture of several botanical species) against PD, by using multiomics analyses. Globally, low back pain is the principal cause of years lived with incapacity. Herein, Huang and Xie reported a Mendelian randomization study in order to evaluate whether dried fruit consumption prevents low back pain.

Cruciferous vegetables (CV), such as Portuguese cabbage, Brussels sprouts, cauliflower and broccoli, are essential components of PBD. Considering the intense debate on the association of consumption of CV with cancer prevention, Yu et al. summarized and discussed the evidence on the possible link between CV intake and bladder cancer risk.

Vitamin E is a fat-soluble antioxidant vitamin, commonly found in nuts, seeds, fruits, vegetables, and edible oils. Herein, Zhang and Yi present a systematic review on the multiple health benefits of this essential nutrient, namely in the prevention and management of several types of cancers, cardiovascular and neurodegenerative diseases.

We are currently facing an unprecedented level of diet-related diseases (1, 2). Yet, some clinicians seem to ignore the potential benefits of a healthy and well-balanced diet, rich in fruits, vegetables and herbs, and quickly prescribe drugs before giving patients a chance to correct their illnesses through shifts in their dietary habits and exercise regime. Therefore, “*Fruits, Vegetables and Herbs: Medicinal Chemistry, Metabolic and Health Effects*” is a timeless and crucial Research Topic, supported by novel and reviewed data analyzed in the papers included in this Research Topic. However, there are still many aspects to be clarified and understood in the amazing world of Fruits, Vegetables and Herbs.

## Author contributions

BMS, LR, CVV, AV, and MBPPO participated in the design of the manuscript, analyzed the bibliographic data, collected them, and drafted the manuscript. All authors critically revised the manuscript, contributed to the article, and approved the submitted version.
